# Genetic architecture and genomic prediction for yield, winter damage, and digestibility traits in timothy (*Phleum pratense* L.) using genotyping-by-sequencing data

**DOI:** 10.1007/s00122-025-04860-9

**Published:** 2025-03-18

**Authors:** N. Vargas Jurado, H. Kärkkäinen, D. Fischer, O. Bitz, O. Manninen, P. Pärssinen, M. Isolahti, I. Strandén, E. A. Mäntysaari

**Affiliations:** 1 Natural Resources Institute Finland, Luke, 31600 Jokioinen, Finland; 2 Boreal Plant Breeding Ltd., 31600 Jokioinen, Finland

## Abstract

**Key message:**

Accurate prediction of genomic breeding values for Timothy was possible using genomic best linear unbiased prediction.

**Abstract:**

Timothy (*Phleum pratense* L.) is a grass species of great importance for Finnish agricultural production systems. Genotyping-by-sequencing along with genomic prediction methods offer the possibility to develop breeding materials efficiently. In addition, knowledge about the relationships among traits may be used to increase rates of genetic gain. Still, the quality of the genotypes and the validation population may affect the accuracy of predictions. The objectives of the study were (i) to estimate variance components for yield, winter damage and digestibility traits, and (ii) to assess the accuracy of genomic predictions. Variance components were estimated using genomic residual maximum likelihood where the genomic relationship matrix was scaled using a novel approach. Genomic breeding values were estimated using genomic best linear unbiased prediction in single- and multiple-trait settings, and for different marker filtering criteria. Estimates of heritability ranged from 0.13 ± 0.03 to 0.86 ± 0.05 for yield at first cut and organic matter digestibility at second cut, respectively. Genetic correlations ranged from −0.72 ± 0.12 to 0.59 ± 0.04 between yield at first cut and winter damage, and between digestibility at first and second cuts, respectively. Accuracy of prediction was not severely affected by the quality of genotyping. Using family cross-validation and single-trait models, predictive ability ranged from 0.18 to 0.62 for winter damage and digestibility at second cut, respectively. In addition, validation using forward prediction showed that estimated genomic breeding values were moderately accurate with little dispersion. Thus, genomic prediction constitutes a valuable tool for improving Timothy in Finland.

**Supplementary Information:**

The online version contains supplementary material available at 10.1007/s00122-025-04860-9.

## Introduction

Timothy (*Phleum pratense* L.) is an important perennial grass species widely used in cool and humid climates across the Nordic countries, central Europe, northeastern and northwestern North America, Russia, and Japan (Berg et al. 1996). Due to its high yield, tolerance to winter conditions in northern latitudes, and high nutritional value, Timothy is commonly used for feeding livestock, either alone or in combination with other grasses and legumes (Niemeläinen et al. 2004), and is offered fresh or preserved as hay or silage (Bélanger et al. 2001). In addition, in Finland, Timothy has been used as a cover crop to reduce bare soil conditions after the production of cash crops such as oats, barley, and peas (Peltonen-Sainio et al. 2022). As such, Timothy cultivation plays an important economic and environmental role in Finnish production systems.

With the intent of improving economically important traits such as yield, digestibility, winter survival, and disease resistance, selection strategies for Timothy have focused on traditional methods, such as progeny testing in multiple environments (Bélanger et al. 2001). Therefore, development of new varieties has been a lengthy and an expensive process. The use of genomic information combined with performance records in the framework of genomic selection (**GS**) provides an alternative to increase the rate of genetic change and accuracy of selection (Meuwissen et al. 2001). Timothy is a hexaploid (2*n* = 6*x* = 42), outcrossing, wind pollinated species (Cai and Bullen 1994; Tanhuanpää and Manninen 2012), and cultivars are usually open pollinated populations. However, its genomic constitution (autohexaploid vs. allohexaploid) is still unclear (Cai and Bullen 1994; Cai et al. 2003), and its reference genome is not yet available. This creates additional challenges for genomic prediction methods (e.g., genome-wide association studies) that rely on a map of the genome. However, genotype-by-sequencing (**GBS**) methods provide an alternative to obtain genomic information in a cost-effective manner (Gorjanc et al. 2015) without the explicit need for a reference genome. Still, challenges remain due to low sequencing depths or allelic biases which may affect predictions of genetic merit (Gerard et al. 2018; de Bem Oliveira et al. 2020).

Knowledge about the genetic architecture of traits of economic importance in Timothy is limited. The heritability of several nutritive quality traits of Timothy clones, including organic matter digestibility and crude protein content, has been previously estimated (Ashikaga et al. 2008) for Japanese populations. However, for the local Finnish (or European) populations and for many other traits of economic importance such information is not yet available. Information about the genetic architecture of traits is not only important for selection decisions but estimates of variance components are also necessary for genomic evaluation. Moreover, determining relationships among traits, whether favorable or unfavorable, is crucial for the development of selection indices (Cerón-Rojas and Crossa 2018). Indices that combine estimates of the genetic merit of several traits along with economic weights can be used to increase rates of genetic gain for sets of traits with favorable correlations while simultaneously limiting changes in traits with unfavorable or antagonistic correlations (Batista et al. 2021). Also, indices can be tailored for specific environments thus making efficient use of the genetic resources available (Louw 1990).

Genomic best linear unbiased prediction models (**GBLUP**) using GBS data have been recently applied to Timothy (Kovi et al. 2019, 2021) and other grass species (Guo et al. 2018; Fiedler et al. 2018). Despite the successful application of genomic prediction, estimates of variance components or genomic breeding values (**GEBV**) from one population do not necessarily generalize to a different population. In addition, the accuracy of GEBV may depend on how the training and validation sets were selected (Kovi et al. 2021). Furthermore, when using GBS data in polyploid species, factors related to the quality of sequencing such as read depth and call rate, and bias due to overdispersion may affect the accuracy of predictions as the genomic relationship matrix may be impacted (Cericola et al. 2018; Guo et al. 2018). Therefore, validating GEBV resulting from genomic evaluations of a Finnish Timothy population where marker information is obtained from GBS data is of paramount importance. The objectives of the current study were to (i) estimate the narrow-sense heritability and genetic correlations for traits of economic importance in Finnish Timothy, and (ii) determine the accuracy of GEBV prediction by using cross-validation through a forward-prediction strategy.

## Materials and methods

### Sequencing and genotypes

Variant data was obtained by LGC Genomics, Berlin, Germany, using double digest restriction associated DNA sequencing (ddRAD), a Genotyping-by-sequencing (GBS) method to reduce the complexity of the genome often used for non-model species with no prior or little genomic knowledge (Peterson et al. 2012). However, due to the lack of a published reference genome for Timothy, a “mock” reference genome was constructed from the ddRAD data from six individuals that represented diverse genetic backgrounds. These six samples were also used to determine the most efficient combination of restriction enzymes as measured by the number of variants produced. As such, it was determined that a sequencing depth of 1.5 million read pairs per sample was sufficient. The combination of restriction enzymes Pstl-ApeKl resulted in the highest number of identified variants. After demultiplexing of the library groups, sequencing adapter remnants from all reads were clipped and reads with a final length < 20 bases were discarded. Following this step, a quality trimming was applied, where reads containing “N” bases were removed and reads were trimmed at the 3’-end to a minimum Phred quality score of 20 over a window of ten bases. The quality trimmed reads were then aligned against the cluster reference using Bowtie 2 (Langmead and Salzberg 2012; version 2.2.3) and variants were called with FreeBayes (Garrison and Marth 2012; version 1.0.2–16) where further filtering criteria were applied (Supplemental File). The resulting variant information was provided in the variant call format (**VCF**) text file.

A total of *n* = 1764 mother clones were genotyped using the ddRAD method described above. Out of these, 107 mothers were genotyped multiple times (two to five) such that the repeated samples could be used to provide a measure of the consistency of the sequencing approach. Read depth information extracted from the associated VCF files included the number of reads for the reference (${R}_{A}$) and alternate (${R}_{B}$) alleles for each of the genotyped individuals. In addition, a single nucleotide polymorphism (**SNP**) marker was considered missing if both ${R}_{A}$ and ${R}_{B}$ were zero. In contrast to SNP chip data, where discrete allele counts are available (i.e., allele dosage), the current study used allele frequencies instead. Thus, genotypes were defined as (Guo et al. 2018):

1 $${f}_{ij}=\dfrac{{R}_{A_{ij}}}{{R}_{A_{ij}}+{{R}_{B_{ij}}}}$$

where ${f}_{ij}$ was the estimated allele frequency of the reference allele for individual *i* (*i* = 1, …, *n*) and marker *j* (*j* = 1, …, *m*), and ${R}_{A_{ij}}$ and ${R}_{B_{ij}}$ were the read depths of the reference and alternate alleles, respectively*.* As defined in (1), ${f}_{ij}$ were continuous variables (or continuous genotypes) on the interval [0, 1]. The number of SNP markers (*m*) depended on the filtering criteria described below. Also, in cases where an individual was sequenced multiple times, their allele frequencies were averaged into one allele frequency.

Due to the use of the ratio in (1), small or large values of the number of reads may lead to biased estimates of allele frequency and thus impact genomic predictions. As such, the impact of different filtering criteria on predictions was tested. In addition to minor allele frequency (**MAF**) ≥ 0.05, markers were filtered (removed) based on their average minimum and maximum number of total reads (${R}_{T}$), and four levels of call rate (100%, 90%, 80%, and 70%). The minimum and maximum average number of reads were set to 10 or 20 and 100 or 200, respectively. Both criteria affected the number of markers available for later use in genomic prediction (Table 1). Before filtering and across all samples there were a total of 280 808 common markers. For this set of markers, call rate ranged from 100.0 to 2.6% with a mean (SD) of 72.4% (24.4%), and there were 222 063 markers (or 79.1% of all markers) with a call rate of at least 70%. Missing SNPs for an individual were imputed using the marker average.

**Table 1:** Number of markers according to different filtering criteria including, minimum and maximum average read depth (RD), and call rate (%)

Minimum RD	Maximum RD	Call rate (%)	Markers
20	100	100	2883
		90	26,452
		80	29,587
		70	30,692
	200	100	4525
		90	29,353
		80	32,488
		70	33,593
10	100	100	2887
		90	38,152
		80	48,802
		70	54,397
	200	100	4529
		90	41,053
		80	51,703
		70	57,298

**Fig. 1:**
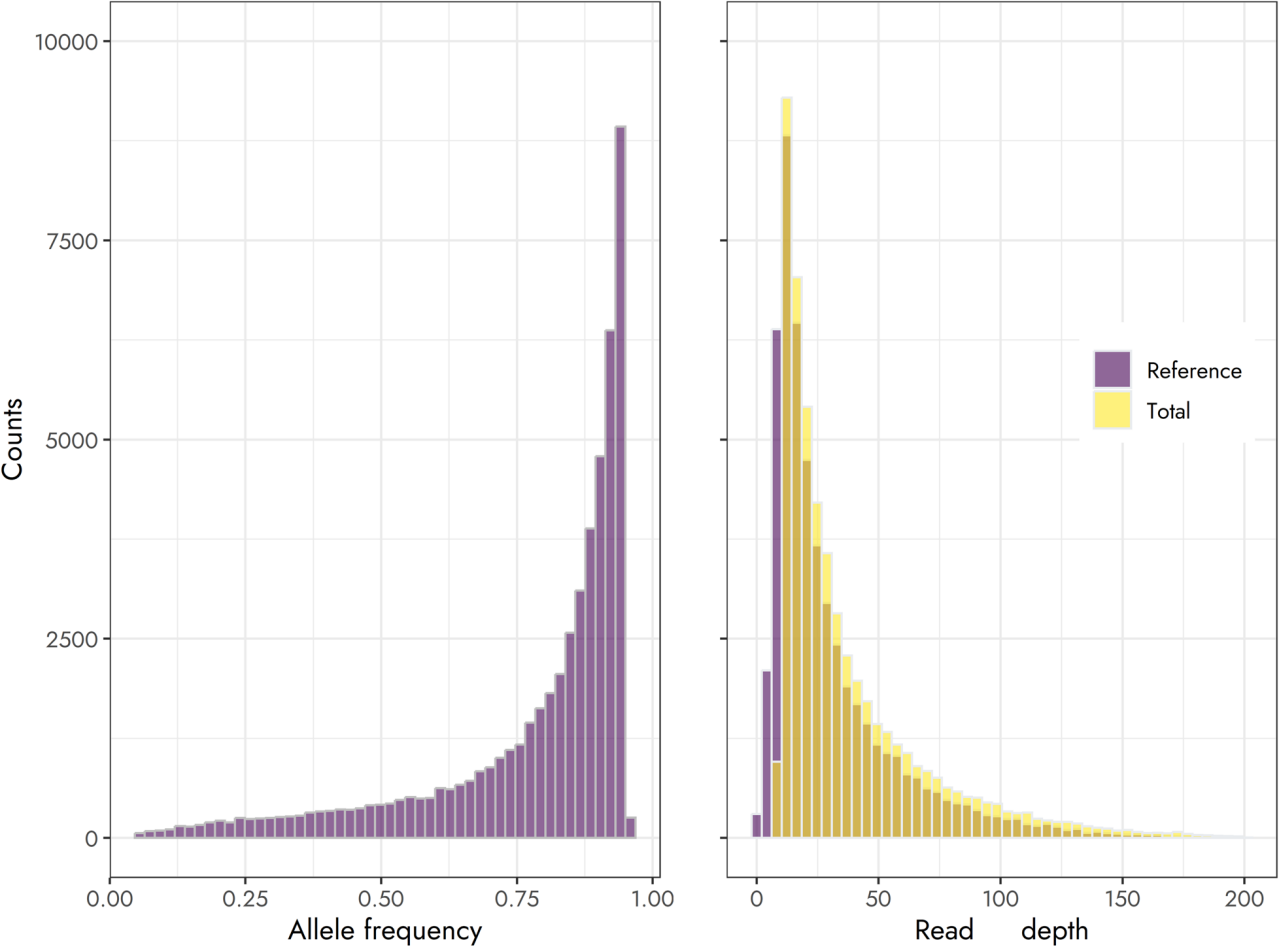
Distribution of allele frequency (for the reference allele) and read depth for Timothy (*Phleum pratense* L.) clones obtained from genotyping-by-sequencing data

After filtering based on MAF, a minimum and maximum read depth of 10 and 200, respectively, and for a call rate of 70% the number of markers was *m* = 58,816. Averaged over markers, the mean total read depth was 37.88 (SD 33.09), ranging from 4.92 to 220.72, while the mean allele frequency was 0.77 (SD 0.20). The distribution of read depth and allele frequencies for the genotyped mother clones is presented in Fig. 1. In addition, the correlation between repeated samples for mother clones genotyped multiple times was, on average, 0.85 (SD 0.10).

### Genomic relationships and scaling factors

The genomic relationship matrix (**G**; $n\times n$) was constructed as (Van Raden 2009):

2 $$\mathbf{G}=\dfrac{\left(\mathbf{F}-\mathbf{1}_{n}\overline{\mathbf{f}}^{\prime}\right){\left(\mathbf{F}-\mathbf{1}_{n}\overline{\mathbf{f}}^{\prime}\right)}^{\prime}}{h}$$

where **F** was the *n* by *m* matrix of genotypes, $\overline{\mathbf{f} }=\left[{\overline{f} }_{1},\dots ,{\overline{f} }_{m}\right]^{\prime}$ was an *m* by 1 vector of mean allele frequencies, ${1}_{n}$ was an *n* by 1 vector of ones, and $h$ was a scaling factor. In the current work three scaling factors and their impact on **G** were studied (i) a naïve approach with a scaling factor derived from allele frequencies, (ii) the de-biasing strategy of Cericola et al. (2018), and (iii) a novel approach based on the Beta-Binomial distribution. The naïve approach used a scaling factor defined as (Guo et al. 2018):

3 $$h=\frac{1}{k}\sum_{j=1}^{m}{\overline{f} }_{j}\left(1-{\overline{f} }_{j}\right)$$

where *k* was the ploidy of an individual (assumed to be 6 for Timothy) and ${\overline{f} }_{j}$ was the mean allele frequency of marker *j*. Due to the low number of reads or bias in the sequencing reads, using (3) as a scaling factor may inflate the diagonal elements of **G** and subsequently the resulting GEBV. The de-biasing strategy by Cericola et al. (2018), on the other hand, addressed this inflation by assuming that the reads for a given allele followed a Binomial distribution:

$${R}_{{A}_{{ij}}}\sim \text{Binomial}\left({R}_{{T}_{{ij}}},{p}_{j}\right)$$

where $i$ indexed over individuals and $j$ indexed over markers, ${R}_{{A}_{{ij}}}$ was the number of reads for the reference allele, ${R}_{{T}_{{ij}}}={R}_{{A}_{{ij}}}+{R}_{{B}_{{ij}}}$ was the total number of reads, and ${p}_{j}$ was the probability of sampling the reference allele, assumed to be constant. Under these assumptions, the mean and variance of the sequencing reads were $\mathbb{E}\left[{R}_{{A}_{{ij}}}\right]={R}_{{T}_{{ij}}}{p}_{j}$ and $\mathbb{V}\text{ar}\left[{R}_{{A}_{{ij}}}\right]={R}_{{T}_{{ij}}}{p}_{j}\left(1- {p}_{j}\right)$ such that the mean and variance of the resulting allele frequencies ${f}_{{ij}}={R}_{{A}_{{ij}}}/{R}_{{T}_{{ij}}}$ were $\mathbb{E}\left[{f}_{{ij}}\right]={p}_{j}$ and $\mathbb{V}\text{ar}\left[{f}_{{ij}}\right]={p}_{j}\left(1-{p}_{j}\right)/{R}_{{T}_{{ij}}}$, respectively. Using these moments and a Normal distribution approximation, Cericola et al. (2018) proposed to correct the diagonals of **G** by ${G}_{{ii}}={\widetilde{G}}_{{ii}}\left(1-{w}_{i}\right)$, where ${\widetilde{G}}_{ii}$ and ${G}_{ii}$ were the biased and the corrected *i*^th^ diagonal element of **G**, respectively, ${w}_{i}=\left(k-1\right)/\left({\overline{R} }_{{T}_{i}}+k-1\right)$ was an adjustment factor, and ${\overline{R} }_{{T}_{i}}$ was the average (total) read depth for an individual (averaged across markers).

In the current study a novel approach, denoted as the Beta-Binomial model, was considered which addressed the bias (overdispersion) in GBS data by assuming that sequencing reads at locus *j* can instead be modeled using the following hierarchy:

4 $$\begin{gathered} R_{{A_{{{{ij}}}} }} | p_{{{{ij}}}} \sim {\text{Binomial}}\left( {R_{{T_{{{{ij}}}} }} ,p_{{{{ij}}}} } \right) \hfill \\ p_{{{{ij}}}} \sim {\text{Beta}}\left( {\mu_{j} , \tau_{j} } \right) \hfill \\ \end{gathered}$$

where ${\mu }_{j}$ and ${\tau }_{j}$ were location and dispersion parameters of the Beta distribution, respectively, such that $\mathbb{E}\left[{p}_{{ij}}\right]={\mu }_{j}$ and $\mathbb{V}\text{ar}\left[{p}_{{ij}}\right]={\mu}_{j}\left(1-{\mu }_{j}\right){\tau }_{j}$. Above, ${\mu }_{j}$ can be considered the underlying “true” allele frequency for locus *j*. As specified in (4), ${p}_{ij}$ could vary across individuals but the location and dispersion parameters were common to all individuals at a given locus. Conditional on ${p}_{ij}$, the expected value and variance of the sequencing reads were the same as in those for the Binomial model. However, for the hierarchical model (4), the marginal mean and variance of the sequencing reads were instead $\mathbb{E}\left[{R}_{{A}_{{ij}}}\right]={R}_{{T}_{{ij}}}{\mu }_{j}$ and $\mathbb{V}\text{ar}\left[{R}_{{A}_{{ij}}}\right]={\mu }_{j}\left(1-{\mu }_{j}\right)\left[1+\left({R}_{{T}_{{ij}}}-1\right){\tau }_{j}\right]$ which can be recognized as the first two moments of a Beta-Binomial distribution. Similarly, the mean and variance of the estimated allele frequencies for the Beta-Binomial model were given by $\mathbb{E}\left[{f}_{{ij}}\right]={\mu }_{j}$ and $\mathbb{V}\text{ar}\left[{f}_{{ij}}\right]={\mu }_{j}\left(1-{\mu }_{j}\right)\left[1+\left({R}_{{T}_{{ij}}}-1\right){\tau }_{j}\right]/{R}_{{T}_{{ij}}}$, respectively. Thus, the assumed uncertainty in the underlying frequency of a marker (${p}_{{ij}}$) was propagated to the estimated allele frequency (${f}_{{ij}}$) as observed by a larger variance.

**Fig. 2:**
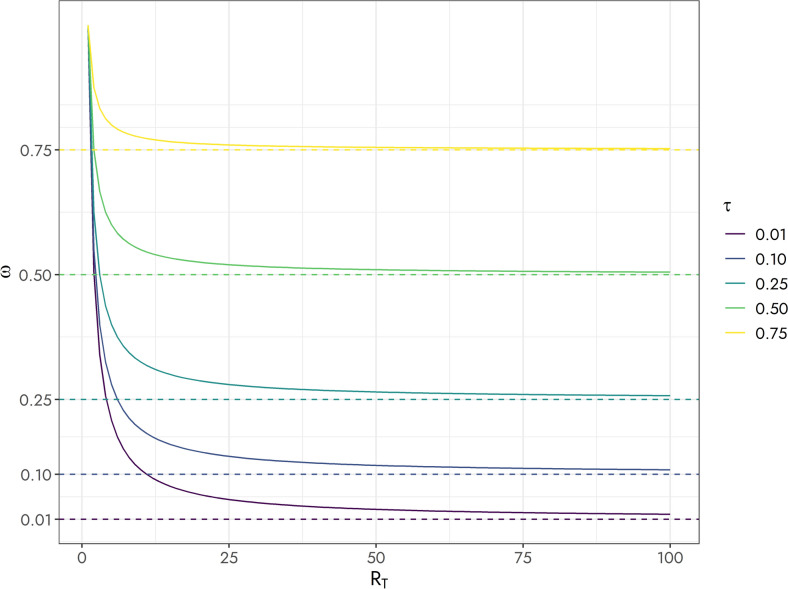
Heterogeneity factor ($\omega$) for the Beta-Binomial model as a function of total read depth ($R_{T}$) and overdispersion parameter ($\tau$)

The scaling factor for **G** proposed in the current work was then given by:

5 $$h=\sum_{j=1}^{m}{\overline{f} }_{j}(1-{\overline{f} }_{j})\frac{\left[1+\left({\overline{R} }_{{T}_{j}}-1\right){\widehat{\tau }}_{j}\right]}{{\overline{R} }_{{T}_{j}}}$$

where the mean allele frequency for marker *j* $\left({\overline{f} }_{j}\right)$ was used as an estimate of the location parameter ${\mu }_{j}$, ${\widehat{\tau }}_{j}$ was an estimate of the overdispersion parameter, and ${\overline{R} }_{{T}_{j}}$ was the mean read depth at locus *j*, averaged across individuals. The scaling factor (5) can be written more generally as $h=\sum_{j=1}^{m}{\overline{f} }_{j}\left(1-{\overline{f} }_{j}\right){\omega }_{j}$ where ${\omega }_{j}=\left[1+\left({\overline{R} }_{{T}_{j}}-1\right){\widehat{\tau }}_{j}\right]/{\overline{R} }_{{T}_{j}}$ was a heterogeneity factor (Williams 1982). When ${\widehat{\tau }}_{j}$ was zero, ${\omega }_{j}$ was equal to $1/{\overline{R} }_{{T}_{j}}$ and its value decreased for increasing ${\overline{R} }_{{T}_{j}}$. On the other hand, if ${\widehat{\tau }}_{j}>0$ and ${\overline{R} }_{{T}_{j}}$ was large, then ${\omega }_{j}$ was almost entirely determined by the overdispersion parameter ${\tau }_{j}$ (Fig. 2). Unlike the de-biasing approach of Cericola et al. (2018), where the adjustment factor was independent of the allele frequency, the scaling factor (5) depends on the overdispersion factor estimated at each locus. Also, note that in (5) the ploidy number is absent because it is a function of the heterogeneity factor. Estimates of ${\tau }_{j}$ were obtained by maximizing the logarithm of the Beta-Binomial likelihood using the optim function in R (R Core Team 2024) with the L-BFGS-B algorithm. Other options for estimating the overdispersion parameter include the quasi-likelihood approach (weighted least squares) of Williams (1982) or Bayesian approaches.

### Field trials and performance records

Timothy (*Phleum pratense* L.) lines evaluated were developed and provided by Boreal Plant Breeding Ltd. The data contained 11,375 performance records corresponding to the progeny of the 1764 genotyped mother clones collected from 2001 to 2023, with an average of approximately 495 records per year. Between 1 and 4 progeny crosses corresponding to approximately 194 (SD 101) mothers were evaluated each year and in an average of 4 locations including southern and northern Finland, Czech Republic, Latvia, and Canada. Progeny lines were the result of top- and poly-crosses. In a top-cross a every mother clone is intermated to the same pollinator variety or other population, while in a poly-cross every mother clone is intermated to all other mother clones in the poly-cross field.

The traits included yield at first, second, and third cut (kg DM/ha), winter damage (%), and digestible organic matter in dry matter (D-value; %; Nousiainen et al. 2003) at first and second cut. Test plots were harvested using an experimental harvester where grass yield was measured, and representative samples were taken for laboratory analysis. Cuttings were made in silage stage, at first cut in growth stages 3.1 – 3.3 according to Moore (Moore et al. 1991). In Finland the first cut was normally made between 10 and 17th of June, the second cut six weeks after the first cut and the third cut in late August/beginning of September. Winter damage was defined as the proportion of deceased individuals in a test plot in the early spring on the second and third year after sowing. D-value was measured with FOSS DS 2500 near-infrared spectroscopy (NIRS) analyzer. D-value of the calibration samples was calculated as (100 – ash content) × OM digestibility, where OM (organic matter) digestibility was assessed by cellulase digestibility, and the ash content of the samples was determined as the proportion of weight remaining after placing the samples in a furnace at 550 °C for 6 h (Rinne and Nykänen 2000).

Prior to this study, performance records were corrected for spatial effects. The trial design in those field trials was a row-column design with two replications. The statistical model used can be described as:

$${y}_{rcl}=\mu +{\theta }_{r}+{\phi }_{c}+{\eta }_{l}+{\epsilon }_{rcl}$$

where $\mu$ was the overall mean, ${\theta }_{r}$, was the effect of row block *r*, ${\phi }_{c}$ was the effect of column block *c*, and ${\eta }_{l}$ was the effect of treatment *l*. Statistical analyses were done with SPSS (IBM SPSS Statistics, version 27, 2019), and the best model was selected based on Akaike's information criterion (AIC). In addition, the performed quality control included removal of outliers. The best linear unbiased estimators obtained after pre-correction were used as the dependent variable (traits) in the subsequent genomic prediction models. Summary statistics including mean, SD, and number of records for these traits are provided in Table 2.

**Table 2:** Summary statistics including, number of records, mean, standard deviation, minimum, and maximum for Timothy (*Phleum pratense* L.) traits used in the genomic evaluation

Trait	*N*	Mean	SD	Min	Max
Yield at 1st cut (kg/ha)	11,192	6460.7	2511.7	354.0	18,476.0
Yield at 2nd cut (kg/ha)	11,214	4159.8	1634.2	269.0	11,224.0
Yield at 3rd cut (kg/ha)	7745	2623.8	978.4	404.0	6675.0
Winter damage (%)	3052	12.1	12.1	−3.0	79.0
D-value at 1st cut (%)	7383	68.2	3.3	54.0	77.9
D-value at 2nd cut (%)	7007	67.6	4.4	53.0	78.9

### Estimation of variance components

The following multiple-trait GBLUP model was used to estimate variance components for the traits mentioned above:

6 $$\mathbf{y}=\mathbf{X}\mathbf{b}+{\mathbf{Z}}_{m}\mathbf{m}+{\mathbf{Z}}_{c}\mathbf{c}+\mathbf{e},$$

where $\mathbf{y}={\left[{\mathbf{y}}_{1},\dots ,{\mathbf{y}}_{T}\right]}^{\prime}$ was the vector of phenotypic records with traits stacked, $\mathbf{b}={\left[{\mathbf{b}}_{1},\dots , {\mathbf{b}}_{T}\right]}^{\prime}$ was the vector of fixed effects including trial by year interaction (210 levels), $\mathbf{m}={\left[{\mathbf{m}}_{1},\dots ,{\mathbf{m}}_{T}\right]}^{\prime}$ was the vector of random genetic effects (GEBV) for mother clones, $\mathbf{c}={\left[{\mathbf{c}}_{1},\dots , {\mathbf{c}}_{T}\right]}^{\prime}$ was the random vector of permanent environmental effects (progeny ID within trial, 5601 levels), and $\mathbf{e}=\left[{\mathbf{e}}_{1} ,\dots ,{\mathbf{e}}_{T}\right]^{\prime}$ was the residual. The block-diagonal matrices $\mathbf{X}={\oplus }_{i=1}^{T} {\mathbf{X}}_{i}$, ${\mathbf{Z}}_{m}={\oplus }_{i=1}^{T}{\mathbf{Z}}_{{m}_{i}}$, and ${\mathbf{Z}}_{c}={\oplus }_{i=1}^{T}{\mathbf{Z}}_{{c}_{i}}$ related records to the fixed, additive, and permanent environmental effects, respectively, and $\oplus$ denotes the direct sum of matrices. In addition, *T* = *6* was the number of traits. Furthermore, it was assumed that $\mathbf{m}\sim {N}\left(\mathbf{0}, {\mathbf{G}\otimes \mathbf{V}_{M}}\right)$,$\mathbf{c}\sim {N}\left(\mathbf{0}, {\mathbf{I}\otimes \mathbf{V}_{C}}\right)$, and $\mathbf{e}\sim {N}\left(\mathbf{0}, {\mathbf{I}\otimes \mathbf{V}_{E}}\right)$ where $\otimes$ denotes the Kronecker (direct) product, **G** was the genomic relationship matrix calculated using the Beta-Binomial scaling factor (defined in (2) and (5)),${\mathbf{V}}_{M}$,${\mathbf{V}}_{C}$, and ${\mathbf{V}}_{E}$ were the 6 by 6 mother, permanent, and residual (co)variance matrices among traits, respectively, and${{\sigma }_{M_k}^{2}}$,${{\sigma }_{C_k}^{2}}$, and ${{\sigma }_{e_k}^{2}}$ correspond to their *k*^th^ diagonal element. Variance components for model (6) were estimated using genomic Monte-Carlo (**MC**) Expectation–Maximization REML (**EM**-**REML**) in MiX99 (Vuori et al. 2006; Matilainen et al. 2019) with 10 MC samples per round. Convergence was assumed when the round-to-round change in the variance component estimates was less than$1\times {10}^{-11}$. Standard errors for (co)variance parameters were calculated with 200 additional MC samples after convergence.

Narrow-sense heritability $({h}_{k}^{2}$) and repeatability (${c}_{k}^{2}$) for trait $k=1,\dots ,6$ were calculated as (Legarra 2016; Becker 1992):

$${h}_{k}^{2}=\frac{\overline{\text{diag }\left(\mathbf{G}\right)}\times {\sigma }_{{A}_{k}}^{2}}{{\sigma }_{{M}_{k}}^{2}\times \overline{\text{diag }\left(\mathbf{G}\right)}+{\sigma }_{{C}_{k}}^{2}+{\sigma }_{{e}_{k}}^{2}}, {c}_{k}^{2}=\frac{\overline{\text{diag }\left(\mathbf{G}\right)}\times {\sigma }_{{A}_{k}}^{2}+{\sigma }_{{C}_{k}}^{2}}{{\sigma }_{{M}_{k}}^{2}\times \overline{\text{diag }\left(\mathbf{G}\right)}+{\sigma }_{{C}_{k}}^{2}+{\sigma }_{{e}_{k}}^{2}}$$

where ${\sigma }_{{A}_{k}}^{2}=4{\sigma }_{{M}_{k}}^{2}$ corresponded to the additive variance for trait *k* and $\overline{\text{diag }\left(\mathbf{G}\right)}$ was the average of the diagonal elements of **G** calculated using the Beta-Binomial method. Given that the mother variance (${\sigma }_{{M}_{k}}^{2}$) explains only ¼ of the genetic variance of the progeny, it was multiplied by 4 (Becker 1992) to obtain the additive variance (${\sigma }_{{A}_{k}}^{2}$). In addition, phenotypic and genetic correlations among the traits described above were calculated as:

$${{r}_{l_{jk}}}=\frac{{\sigma }_{{l}_{jk}}}{\sqrt{{\sigma }_{{l}_{jj}}^{2}{\sigma }_{{l}_{kk}}^{2}}}$$

where ${{r}_{l_{jk}}}$ was the correlation between traits *j* and *k* for the *l* effect (for *l* = *A, P* for additive and phenotypic, respectively), and the additive variance and phenotypic variances were defined as ${\mathbf{V}}_{A}=4{\mathbf{V}}_{M}$ and ${\mathbf{V}}_{P}={\mathbf{V}}_{M}+{\mathbf{V}}_{C}+{\mathbf{V}}_{E}$, respectively

### Validation of GEBV

Estimation of GEBV was performed using both single-trait and multi-trait GBLUP models (6). The single-trait model was similarly defined as:

7 $$\mathbf{y}=\mathbf{X}\mathbf{b}+{\mathbf{Z}}_{m}\mathbf{m}+{\mathbf{Z}}_{c}\mathbf{c}+\mathbf{e},$$

where $\mathbf{y}$ was the vector of phenotypic records for a given trait, $\mathbf{X}$, ${\mathbf{Z}}_{m}$, and ${\mathbf{Z}}_{c}$ corresponded to the design matrices for the fixed, mother, and permanent environmental effects, respectively. In addition, $\mathbf{m}\sim N\left(\mathbf{0},{\sigma }_{M}^{2}\mathbf{G}\right)$, $\mathbf{c}\sim N\left(\mathbf{0},\mathbf{I}{\sigma }_{C}^{2}\right)$, and $\mathbf{e}\sim N\left(\mathbf{0},\mathbf{I}{\sigma }_{e}^{2}\right)$ were, as before, the mother, permanent environmental, and residual effects, the variances ${\sigma }_{M}^{2}$, ${\sigma }_{C}^{2}$, and ${\sigma }_{e}^{2}$ were the appropriate diagonal element of their corresponding matrix (${\mathbf{V}}_{M}$, ${\mathbf{V}}_{C}$, or ${\mathbf{V}}_{E}$) estimated from the multiple-trait model (6), and $\mathbf{I}$ was an identity matrix. In the current work, prediction, and validation of GEBV was not performed for specific environments or locations but across all locations. However, to account for potential differences in performance among environments (or locations), both models (6) and (7) included the trial by year and the permanent environmental effects, which incorporate trial location in their definition. Because the **G** matrix scaled using the naïve and Beta-Binomial model are the same up to a constant multiplier (e.g., all elements of **G** are multiplied by a scalar) no differences are expected in terms of estimates of variance components or the corresponding GEBV. On the other hand, for the debiased approach potential differences may arise. Thus, such impacts are considered in subsequent analyses.

GEBV were validated using two strategies: (i) a family cross-validation, and (ii) a forward-prediction strategy. For both (i) and (ii) two sets of individuals were defined: the estimation set which corresponded to mother clones whose progeny records were used for estimating GEBV using models (6) and (7), and a validation set which corresponded to mother clones whose progeny records were not used in the evaluation but instead were used for calculating predictive ability (and other accuracy measures). However, the genomic information of all mother clones was used in the estimation of GEBV such that the predicted GEBV for individuals in the validation set were derived from the genomic relationships with individuals in the estimation set. For (i), a total of 18 half-sib progeny families were defined such that there were at least 50 mothers, and their progeny, in each family. Thus, there were 18 individual evaluations where the performance records corresponding to the progeny of the mother clones in a given family were not used to estimate their GEBV. The number of mother clones in the validation set ranged from 54 to 262 representing approximately 5% and 23% of all genotyped mothers, and were associated with 48 and 251 individual progenies, respectively. For (ii), on the other hand, performance records from the last four years of recording (2020, 2021, 2022, and 2023) were considered part of the validation set and thus not used in the genomic evaluation (estimation of GEBV). This resulted in 8217 phenotypic records for the estimation set and 3158 for the validation set. That is, about 27% of the records were removed. A total of 338 mother clones were identified that had records in the full data set but did not have records in the estimation set.

For the mothers in the validation set, predictive ability was defined as the correlation between GEBV and mean progeny performance, adjusted for fixed and permanent environmental effects. The impact of the marker filtering criteria previously described on predictive ability was tested by constructing **G** with the marker sets shown in Table 1 and fitting the model (6) or (7). In addition, the validation method described in Legarra and Reverter (2018) was used, where the full data set consisted of the progeny records of all genotyped mothers, while the reduced data set was defined as the progeny records from mothers remaining in the evaluation after removing mothers in a validation group. For the full and reduced sets, GEBV were obtained, and the following criteria were calculated: (i) the correlation between GEBV in the full and reduced sets, and (ii) the slope (or dispersion) and (iii) the coefficient of determination from the regression of GEBV from the full set on the GEBV from the reduced set. To avoid confusion with overdispersion in the read depth of SNPs, the terms over- and under-prediction are used to refer to over- and -under-dispersion of GEBV.

## Results

### Scaling factor

**Fig. 3:**
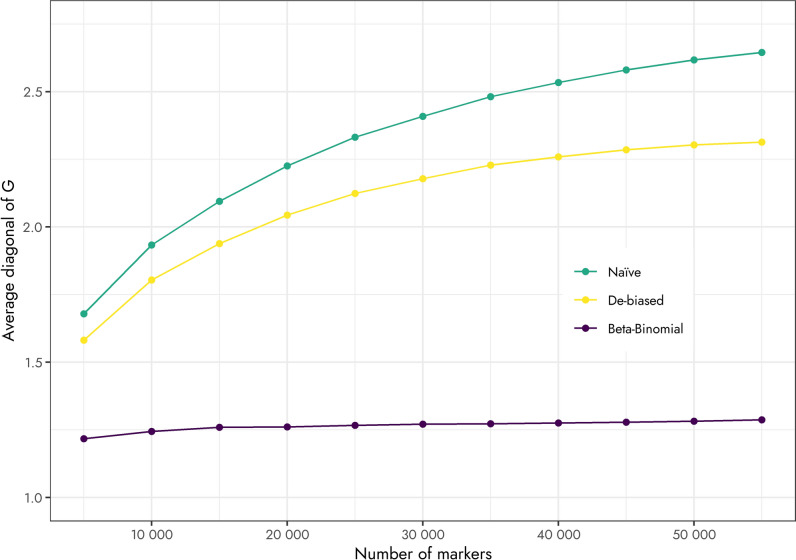
Average diagonal values of **G** scaled under naïve, debiased, and Beta-Binomial model assumption for Timothy clones (*Phleum pratense* L.) sequenced using genotyping-by-sequencing

Averaging across the marker sets (Table 1), the mean of the diagonal elements of **G** was 2.32 ± 0.09, 2.06 ± 0.07, and 1.26 ± 0.01 when scaled using the naïve, debiased, and Beta-Binomial approaches, respectively. In addition, the average of the diagonals of **G** increased along with the number of markers for the naïve and debiased approaches. This was less the case when **G** was scaled using the Beta-Binomial scaling factor (Fig. 3).

Across all markers, the mean estimate of the overdispersion parameter $\widehat{\tau }$ was 0.26 (SD 0.16), with individual values ranging from 0.00 to 0.76. In the same manner, the mean heterogeneity factor ($\widehat{\omega }$) was 0.30 (SD 0.15) with values ranging from 0.01 to 0.77. In addition, the correlation between $\widehat{\omega }$ and $\widehat{\tau }$ was 0.99.

### Estimates of variance components

Estimates of additive variance were 60,342.8 ± 12,184.4, 102,520.8 ± 10,432.4, and 21,229.6 ± 3912.0, for yield at first, second, and third cuts, respectively, 6.60 ± 2.28 for winter damage, and 0.72 ± 0.08 and 1.24 ± 0.12 for D-value at first and second cuts, respectively. Similarly, estimates of permanent environmental variances were 79,075.3 ± 9021.0, 29,488.8 ± 3685.9, and 11,370.6 ± 1988.1, for yield at first, second, and third cuts, respectively, 5.88 ± 1.65 for winter damage, and 0.03 ± 0.02 and 0.10 ± 0.03 for D-value at first and second cuts, respectively. Likewise, estimates of residual variances were 482,725.8 ± 10,220.0, 179,893.3 ± 4114.8, and 82,912.9 ± 2319.9 for yield at first, second, and third cuts, respectively, 35.44 ± 1.84 for winter damage, and 0.89 ± 0.02 and 1.32 ± 0.04 for D-value at first and second cuts, respectively. Lastly, favorable genetic covariances were found among yield traits, ranging from 2331.4 ± 1161.7 to 5256.7 ± 1144.9, between cuts 1 and 3, and between cuts 2 and 3, respectively. Similarly, a smaller covariance of 0.14 ± 0.02 was found among D-value traits. On the other hand, negative covariances ranging from −114.3 ± 31.0 to −0.04 ± 0.01 were found between winter damage and yield at first cut, and between winter damage and D-value at first cut, respectively.

Estimates of heritability ranged from 0.13 ± 0.02 for yield at first cut to 0.86 ± 0.05 for D-value at second cut (Table 3). Estimates of genetic correlations among traits ranged from −0.72 ± 0.12 between yield at first cut and winter damage to 0.59 ± 0.04 between D-value at first and second cut. Phenotypic correlations, on the other hand were smaller in magnitude, ranging from −0.45 ± 0.02 between yield at first cut and winter damage to 0.23 ± 0.01 between yield at second and third cuts.

**Table 3:** Estimates of heritability (diagonal, bold), genetic correlations (lower triangle), and phenotypic correlations (upper triangle) for yield traits (kg/ha), winter damage (%), and D-value (%) in Timothy (*Phleum pratense* L.).

**Trait**	Yield at 1st cut	Yield at 2nd cut	Yield at 3rd cut	Winter damage	D-value at 1st cut	D-value at 2nd cut
Yield at 1st cut	**0.13** (0.03)	0.10 (0.01)	0.11 (0.01)	−0.45 (0.02)	−0.17 (0.01)	0.11 (0.01)
Yield at 2nd cut	0.13 (0.08)	**0.53** (0.04)	0.23 (0.01)	−0.01 (0.02)	−0.04 (0.01)	−0.29 (0.01)
Yield at 3rd cut	0.26 (0.10)	0.45 (0.06)	**0.26** (0.04)	−0.01 (0.03)	0.01 (0.01)	−0.05 (0.02)
Winter damage	−0.72 (0.12)	0.37 (0.11)	−0.09 (0.14)	**0.19** (0.05)	0.19 (0.02)	−0.15 (0.02)
D−value at 1st cut	−0.42 (0.08)	−0.26 (0.04)	0.03 (0.07)	−0.08 (0.10)	**0.80** (0.06)	0.12 (0.03)
D−value at 2nd cut	0.04 (0.08)	−0.64 (0.06)	−0.08 (0.08)	−0.52 (0.11)	0.59 (0.04)	**0.86** (0.05)

Parameter estimates obtained from a multiple-trait model with the genomic relationship matrix scaled using the Beta-Binomial approach. Standard errors of estimates in parentheses

Estimates of repeatability were 0.27 ± 0.02, 0.66 ± 0.04, 0.38 ± 0.04, 0.33 ± 0.05, 0.83 ± 0.05, and 0.91 ± 0.05 for yield at 1st, 2nd and 3rd cuts, winter damage, and D-value at 1st and 2nd cuts, respectively.

### Accuracy of genomic prediction

**Fig. 4:**
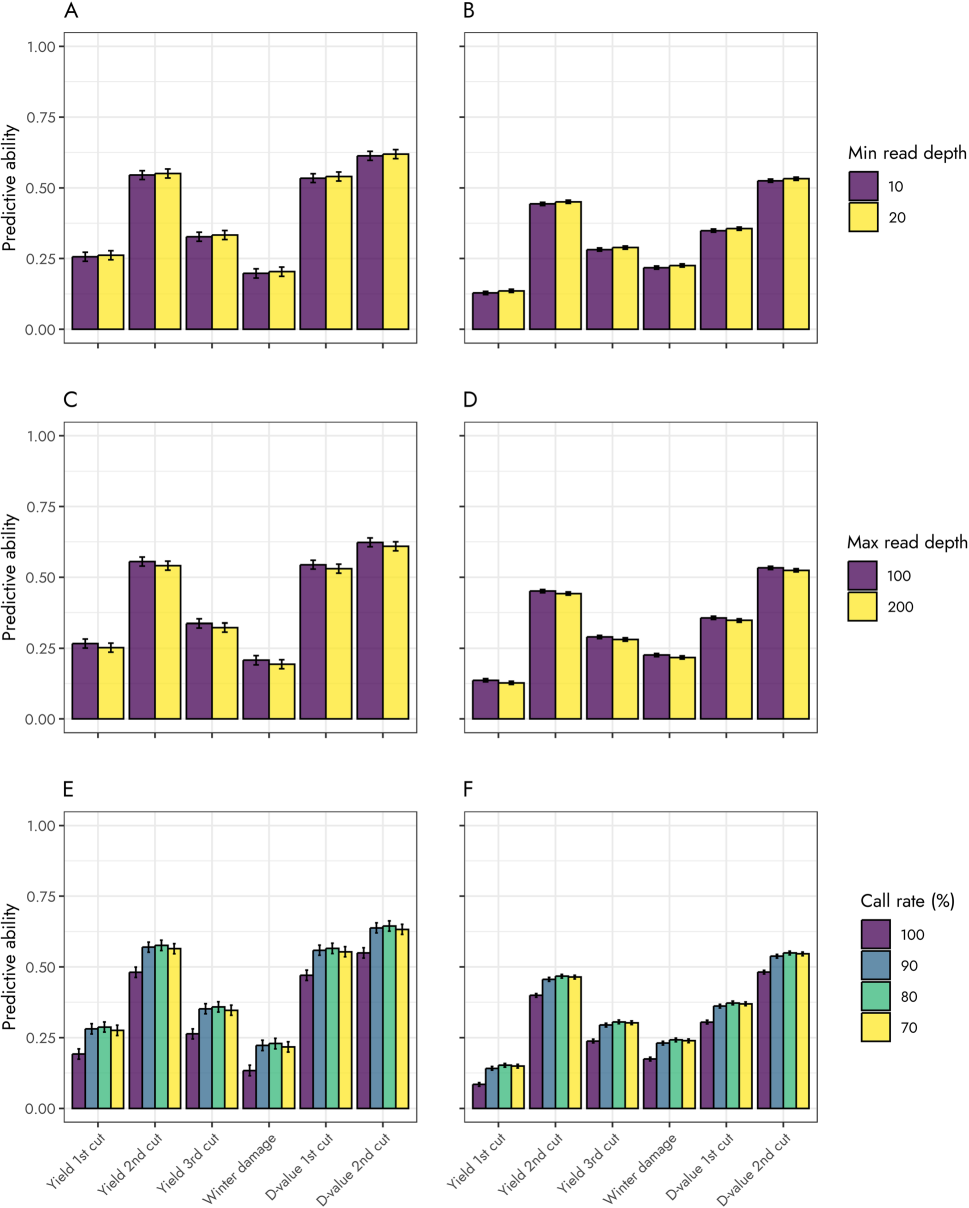
Marginal means for the effect of minimum (top row) and maximum (middle row) read depth, call rate (bottom row) and validation strategy on predictive ability (correlation between genomic breeding value and mean progeny phenotypes adjusted for fixed and permanent environmental effects), averaged over type of model (single- vs multiple-trait), for yield traits (kg/ha), winter damage (%), and D-value (%) in Timothy (*Phleum pratense* L.). Marginal means for family cross-validation in the left column (A, C, E) and for forward prediction on the right column (B, D, F)

Filtering markers based on minimum read depth had little effect on predictive ability for both the family cross-validation and forward-prediction strategies (*P* = 0.31; Fig. 4; Supplementary Table S1). Averaging across traits, marginal means for predictive ability was 0.412 ± 0.002 and 0.418 ± 0.002, and 0.324 ± 0.004 and 0.331 ± 0.004 for minimum read depth of 10 and 20, respectively, for the family cross-validation and forward-prediction strategies, respectively. This represented an increase in predictive ability, with respect to a minimum read depth of 10, of 1.4%, and 1.7%, respectively.

On the other hand, a larger effect was observed when filtering markers on maximum read depth (*P* = 0.014; Supplementary Table S2). Overall, marginal means for predictive ability were 0.422 ± 0.002 and 0.408 ± 0.002, and 0.332 ± 0.004 and 0.323 ± 0.004 for maximum read depth of 100 and 200, respectively, for the family cross-validation and forward-prediction strategies, respectively. These changes in predictive ability, with respect to maximum read depth of 200, represented an increase of 3.4% and 2.8%, respectively. Similarly, call rate had a considerable effect on predictive ability (*P* < 0.01; Supplementary Table S3). Marginal means for predictive ability were 0.348 ± 0.006, 0.437 ± 0.006, 0.444 ± 0.006, and 0.432 ± 0.006 for call rates of 100%, 90%, 80%, and 70% for the family cross-validation approach. Similarly, for the forward-prediction strategy, marginal means for predictive ability were 0.280 ± 0.002, 0.337 ± 0.002, 0.348 ± 0.002, and 0.345 ± 0.002 for call rates of 100%, 90%, 80% and 70%, respectively. The maximum change in predictive ability, compared to a call rate of 100%, represented an increase of 27% and 24% for the cross-validation and forward-prediction strategies, respectively.

Predictive ability decreased when using the debiased approach (*P* < 0.01). Averaging over traits, marginal means for predictive ability were 0.30 ± 0.02 and 0.39 ± 0.02 for the debiased, and Beta-binomial (and naïve) approaches, respectively. This represented a 23% decrease in predictive ability. The decrease in predictive ability ranged from 14 to 48% for winter damage and yield at second cut, respectively.

**Fig. 5:**
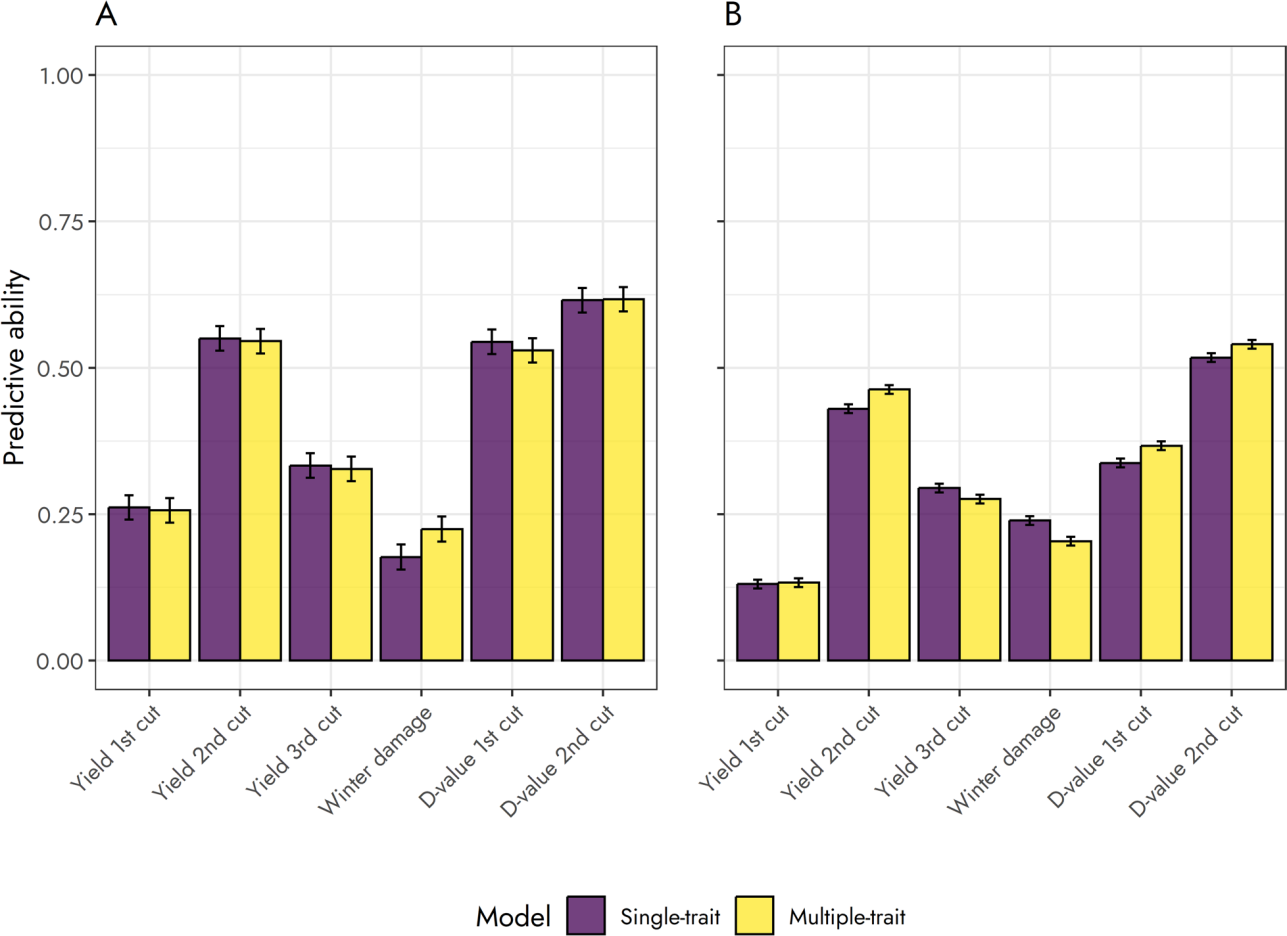
Marginal means for the effect of type of model (single- and multiple-trait) and validation strategy (family cross-validation (A) and forward prediction (B)) on predictive ability (correlation between genomic breeding value and mean progeny phenotypes adjusted for fixed and permanent environmental effects), averaged over minimum and maximum read depth, and call rate, for yield traits (kg/ha), winter damage (%), and D-value (%) in Timothy (*Phleum pratense* L.)

Overall, multiple-trait models resulted similar predictive abilities (*P* = 0.61). Averaging across traits, marginal means for predictive ability were 0.414 ± 0.004 and 0.417 ± 0.004, and 0.325 ± 0.002 and 0.330 ± 0.002 for single- and multiple-trait models, respectively, for the family cross-validation and forward-prediction scenarios, respectively. However, the effect of single- and multi-trait models on predictive ability was, in general, not consistent across traits or validation strategies (Fig. 5; Supplementary Table S4). For the family cross-validation strategy, the use of multi-trait models increased the predictive ability for winter damage by 0.05 (or 27%), while it resulted in a slight decrease (less than 3%) for the remaining traits. For the forward-prediction strategy, on the other hand, fitting the multi-trait GBLUP increased the predictive ability for yield at the second cut, and D-value at the first and second cuts by 0.033, 0.030, and 0.023, respectively.

Additional validation criteria, including correlations between GEBV for the full and reduced sets, slope (or dispersion, ${\beta }_{1})$, and coefficient of determination (*R*^*2*^) from regression of GEBV from the full on the reduced set, for the family cross-validation and the forward-prediction strategies are presented in Table 4. Overall, the correlations were higher for the forward-prediction strategy and for single-trait GBLUP. Multi-trait GBLUP increased predictive ability only for yield at second cut and D-value at first cut and only in the forward-prediction scenario. On the other hand, GEBV accuracy decreased when fitting multi-trait GBLUP, with the largest change in accuracy for D-value at second cut in the family cross-validation scenario.

**Table 4:** Correlation between genomic breeding values (GEBV), slope (or dispersion; $\beta_{1}$), and coefficient of determination (*R*^2^) for GEBV from full and reduced data sets for family cross-validation, and forward-prediction strategies for yield traits (kg/ha), winter damage (%), and D-value (%) in Timothy (*Phleum pratense* L.)

Model	Trait	Family cross-validation			Forward prediction		
		Correlation^a^	$\beta_{1}$^b^	*R*^2b^	Correlation^1^	$\beta_{1}$^b^	*R*^2b^
Single-trait	Yield first cut	0.61	1.01	0.40	0.71	0.95	0.51
	Yield second cut	0.43	0.76	0.20	0.63	0.92	0.40
	Yield third cut	0.61	1.01	0.42	0.57	0.75	0.33
	Winter damage	0.70	1.10	0.52	0.79	1.37	0.63
	D-value first cut	0.56	0.92	0.34	0.55	0.96	0.30
	D-value second cut	0.62	0.96	0.42	0.73	0.99	0.53
Multiple-trait	Yield first cut	0.60	0.98	0.38	0.67	0.94	0.46
	Yield second cut	0.62	0.73	0.20	0.64	0.92	0.41
	Yield third cut	0.59	0.97	0.38	0.57	0.73	0.33
	Winter damage	0.57	0.90	0.35	0.76	1.12	0.58
	D-value first cut	0.52	0.88	0.29	0.59	1.00	0.35
	D-value second cut	0.50	0.82	0.28	0.69	1.03	0.48

^a^Pearson correlation coefficient between GEBV obtained from full and reduced data sets. ^b^Slope and coefficient of determination from regressing GEBV obtained from a full model on GEBV obtained from the reduced model

Dispersion was small, in general, and not consistent across traits or validation strategies, but was less pervasive for single-trait GBLUP models. For the family cross-validation strategy, the largest over-prediction and under-prediction were observed for yield at second cut and winter damage, respectively. However, winter damage changed from under-prediction when using single-trait GBLUP to over-prediction when fitting multi-trait models. Moreover, GEBV were over-predicted for all traits when using multi-trait GBLUP. For the forward-prediction strategy, GEBV for winter damage were under-predicted the most when fitting single-trait GBLUP, which was reduced when using multi-trait models. On the other hand, a large increase in over-prediction was observed for yield at third cut when fitting multiple-trait GBLUP.

In the family cross-validation strategy, *R*^*2*^ values from the regression of GEBV from the full set on the reduced set ranged from 0.20 to 0.52 for yield at second cut and winter damage, respectively. However, for the forward-prediction strategy the ${R}^{2}$ values ranged from 0.33 to 0.63 for yield at third cut and winter damage, respectively. Overall, the ${R}^{2}$ values were larger for the forward-prediction strategy and the single-trait GBLUP models. In the family cross-validation scenario, fitting multi-trait GBLUP reduced the *R*^*2*^ values, while an increase in *R*^*2*^ was observed only for the forward-prediction strategy for yield at second cut and D-value at first cut.

## Discussion

### Genotypes

A similar distribution of read depth to the one presented in our study has been reported for ryegrass (Guo et al. 2018). Due to the skewed distribution of read depth, applying a minimum read depth criterion of 10 instead of 20 increased the number of available markers substantially more than choosing a maximum read depth criterion of 200 instead of 100. While markers at the lower end of the distribution may be affected by low coverage and allelic bias, those at the upper end of the distribution may be outliers that are also affected by allelic bias (Gerard et al. 2018). Therefore, restricting markers to a pre-specified range may be necessary to improve the quality of the available genotypes.

### Scaling factor

To our knowledge, this is the first time that overdispersion has been estimated for GBS data and used to scale **G**. The increase in the mean values of the diagonals of **G** for the naïve (Binomial) and the debiased approach by Cericola et al. (2018) suggest that the marker variance is considerably larger than ${p}_{j}\left(1-{p}_{j}\right)$, and that overdispersion plays a considerable role when sequencing Timothy materials. Indeed, this has been reported by Gerard et al. (2018), where other systematic biases in addition to overdispersion, including sequencing error, allelic bias, and outliers, were modeled to increase the accuracy of genotyping for polyploid species. Similarly, Clark et al. (2019) used a Bayesian model to adjust SNPs for biases by accounting for overdispersion and a contamination rate (i.e., sequencing error). Nevertheless, when calculating genomic relationships within a population, biases may have less of an impact when the number of markers is large (i.e., may be averaged out), such that GEBV and their ranking are less affected by the scaling of **G**.

Because the scaling factors for the naïve and Beta-Binomial approaches are constants (i.e., a single scalar value for all individuals genotyped), the corresponding **G** matrices are also identical (up to a constant). As such, with proper scaling the corresponding estimates of variance and GEBV would also be identical. Such is not the case for the debiased approach as each element of the diagonal of **G** is adjusted (or corrected) differently. While the overall adjustment of the diagonal values of **G** for the debiased approach was small for this Timothy population, the corresponding GEBV were subsequently impacted.

While the main aim of developing the scaling factor was to address the inflation of the diagonals of **G** due to overdispersion in the GBS data, when properly scaled, **G** also provides information about inbreeding (i.e., genomic inbreeding; Villanueva et al. 2021). In our current work the average diagonal of **G** was 1.26, suggesting an inbreeding coefficient of 0.26 (or 26%). While little information related to inbreeding levels of Timothy is available, estimated inbreeding coefficients for ryegrass ranged from 0.27 to 0.65, depending on the type of individual (F1 vs twice selfing of F1 individuals; Harris et al. 2023). However, it should be noted that estimates of heterozygosity using GBS may be unreliable because a site may be regarded as homozygous simply because it was not sequenced sufficiently (Wang et al. 2020).

### Estimates of variance components

To our knowledge, (co)variance estimates for traits of economic importance in Timothy have not been previously reported, further emphasizing the importance of our current work. On the other hand, variance estimates for dry matter yield and disease resistance has been reported for other grass species (Guo et al. 2018). However, due to differences in the definition of traits (e.g., kg/m^2^ vs kg/ha) their reported variances differ substantially to the ones from our study. Thus, parameters from one population may not always (easily) translate from one population to another.

In addition to narrow-sense heritability, information related to broad-sense heritability has been reported for Timothy (Ashikaga et a., 2011) where estimates of genotypic variance were estimated from analysis of variance. Other strategies for estimating broad-sense heritability require estimation of variances due to genotype, year, location, and their interaction, or dominance (and epistatic) effects (Schmidt et al. 2019; Wittenburg et al. 2013). Due to unbalanced trials and the need for correcting for spatial effects, and lack of information on allele dosage in our study, estimates of broad-sense heritability could not be obtained.

Repeatability estimates indicate that the permanent environment of an individual contributes considerably to the variability observed in Timothy. Such contributions were more noticeable for traits with lower heritability (e.g., yield at first cut, winter damage). Because plots may be recorded multiple times in a trial, accounting for permanent environmental effects can help to better separate environmental and additive effects, thereby improving estimates of heritability.

The heritability of yield traits, ranging from 0.11 to 0.44, reflects a considerable environmental effect. For the first cut, performance is determined by how well individuals are established in a field, as well as by the effect of the previous winter after the first year of recording. For the second cut, on the other hand, environmental conditions would be more stable and homogeneous, such that performance depends more on the genetic potential of the individual. Finally, because not every location is suitable for a third cut, and because of changing weather conditions from late summer to fall, performance at this stage is again heavily influenced by the environment. Our estimates differ considerably from those reported by Ashikaga et al. (2016), who reported a broad-sense heritability of 0.79 and 0.91 for dry matter yield at first and second cuts, respectively. These discrepancies, however, likely reflect not only the different populations considered but also the methods used to obtain the estimates as Ashikaga et al. (2016) used partial least-squares regression for predictions and functions of the mean squares derived from analysis of variance to estimate the variance components, and the clones under evaluation had not been selected for nutritive value traits.

The estimate of heritability for winter damage was moderately low suggesting a strong environmental component, and the variability in winter conditions at the locations in which the Timothy materials were tested (e.g., Czech Republic and northern Finland). On the other hand, Ashikaga et al. (2016) reported a broad-sense heritability of 0.66 for winter survival, which was scored on a scale from 1 to 9, with 1 and 9 representing poor and good survival, respectively. Thus, in addition to population differences, winter damage and winter survival may represent different traits, and the differences in heritability may be expected.

Estimates of heritability for digestibility traits (D-value) were high for both cuts, suggesting a stronger genetic component, and the possibility of faster genetic progress for these traits. While not the same traits, Ashikaga et al. (2011) and Ashikaga et al. (2016) reported high heritability estimates for nutritional quality traits, including low-digestible fiber, organic cell wall, and water-soluble carbohydrate fractions, which are strongly associated with digestibility of plant material (Huhtanen and Krizsan 2023). Furthermore, their heritability estimates at the second cut were higher than those at the first cut, which was also the case in our study. Thus, despite potential environmental effects due to possible hot and dry summers, a considerable additive variance still influences digestibility traits in the Finnish Timothy population.

Estimates of additive variance (or heritability) coupled with selection intensity and cycle length (or generation interval), can be used to obtain knowledge about the response to selection (rate of gain) for different breeding strategies (Lush 1937; Cobb et al. 2019). While larger additive variances (and heritability) are associated, in general, with a larger response to selection, faster genetic gains can be achieved by decreasing cycle length (Cobb et al. 2019). Thus, given the estimates of additive variance for yield and digestibility in Timothy, using genomic selection to both reduce the cycle length and increase accuracy of selection can potentially increase the rates of genetic gain.

Positive correlations among yield traits (e.g., yield at second and third cuts), and among D-value traits (e.g., first and second cuts) indicate that selection for one of the traits is likely to have a positive impact on the remaining traits, potentially increasing the rate of genetic progress. Moreover, individual D-value traits were negatively correlated with yield traits (e.g., yield and D-value for the second cut), which is consistent with the results in Claessens et al. (2004) who reported a phenotypic correlation of −0.44 between dry matter yield and true in-vitro digestibility. These negative relationships between digestibility and dry matter yield have been previously reported for Timothy under northern climate conditions (Thorvaldsson et al. 2007; Hyrkäs et al. 2018). On the other hand, a positive genetic correlation between dry matter yield and winter hardiness, defined as the combined effect of tillers per unit after winter damage, was found for ryegrass (Fè et al. 2015). These favorable genetic correlations among yield traits coupled with unfavorable genetic correlations between yield traits and digestibility, and between some yield traits and winter damage reflect the challenging relationships between these traits and environmental conditions. For instance, individuals affected by harsh winter conditions are likely to have poor performance at their first cut of the following season, and individuals who are still in the growing phases when winter conditions arrive are also likely to be impacted such that their subsequent performance next season will also be affected. In the same manner, high yielding individuals may have low digestibility. Thus, indices should be implemented for the selection of suitable materials that combine high yield, digestibility, and tolerance to winter conditions.

### Genomic prediction and validation

Although substantially increasing (or decreasing) the number of available markers, filtering criteria related to minimum and maximum read depth had only a small effect on predictive ability. On the other hand, the larger effect observed due to call rate showed that genomic relationships were more accurately calculated with more markers, despite the potential effect bias and overdispersion. However, with a larger proportion of markers requiring imputation, a slight decrease in predictive ability was observed at the lowest level of call rate. In addition, the lack of a reference genome, lack of allele dosage information (i.e., allele counts), and our use of continuous genotypes (i.e., ratio of number of reads) limits the types of methods that can be used for increasing the accuracy of imputation.

While multi-trait models have shown improvement of predictive ability in ryegrass (Arojju et al. 2020), a clear advantage of multi-trait models was not observed in the current study. In our validations, no phenotypic records were available for genotyped individuals in the validation set, such that predictions of GEBV for these individuals were based entirely on the genomic relationships with other genotyped individuals in the estimation set, and on the genetic relationships among traits. Thus, uncertainty in the estimates of genetic correlations, along with collinearity may lead to poor predictive ability for multiple-trait models.

The discrepancy in predictive ability across traits for the two validation strategies is likely due to differences in the size and structure of the training and validation sets. For the family cross-validation strategy, records are removed (to a certain extent) uniformly across years. On the other hand, a considerable portion of the records were collected in the last three years (2020 to 2022) such that the predictive ability is more affected for the forward-prediction strategy. Overall, the predictive ability for yield traits was similar to that reported by Kovi et al. (2021) for a second generation of full-sib families. These authors reported prediction accuracies ranging from 0.18 for yield at first cut to 0.54 for yield at second cut which are comparable to those found in the current work. Similar predictive abilities have also been reported for dry matter yield in switchgrass and ryegrass (Fiedler et al. 2018; Guo et al. 2018). On the other hand, the accuracy reported by Kovi et al. (2021) for yield at the third cut was much lower (−0.03 to 0.01) than the one obtained in our study. However, the differences in accuracy may be due to a larger validation population (more individuals genotyped) and larger number of phenotypic records in our study. Also, Kovi et al. (2021) used hierarchical clustering to define validation groups as compared to the family cross-validation and forward-prediction strategies in our study.

Predictive ability for winter damage was the lowest for single-trait models in the family cross-validation scenario. An increase in predictive ability for multiple-trait GBLUP was perhaps due to some half-sibs having records for winter damage and other traits, thus increasing the available information for the estimation of GEBV. On the other hand, for the forward-prediction strategy such may have not been the case, leading to a decrease in predictive ability. In addition, in the forward-prediction validation scenario, winter damage was found to have the most under-prediction when using single-trait GBLUP, probably due to the low number of records overall and outside of Finland, and the lower heritability of the trait. Such under-prediction was ameliorated when multi-trait models were used. Thus, multiple-trait models may be more useful when there is interest in improving winter survival.

Overall, the predictive ability for digestibility traits was moderately high, likely due to high heritability and a relatively large number of records in the training population. In switchgrass, in-vitro dry matter digestibility had a predictive ability of 0.69 when cross-validation was performed across the entire dataset (Fiedler et al. 2018). This predictive ability is consistent with that obtained from the family cross-validation in our study. On the other hand, the predictive ability for organic matter digestibility in ryegrass was approximately 0.20 (Arojju et al. 2020) which more closely corresponds to the predictive ability of D-value at first cut in our study for the forward-prediction strategy. For both validation strategies, D-value GEBV were not affected by over- or under-prediction. In addition, single- and multi-trait GBLUP were similarly accurate.

Using the Legarra and Reverter (2018) methodology, moderately high correlations between GEBV from full and reduced sets, along with minimal dispersion (under- or over-prediction), and moderate ${R}^{2}$ values were found for all traits and for both the family cross-validation and forward-prediction methodologies. This suggest that the predictions of GEBV were accurate, likely due to the high number of records and moderate heritability for the traits. Bornhofen et al. (2022) reported similar dispersion values for GEBV of dry matter yield and forage quality traits in ryegrass, ranging from 0.55 to 1.51. Similarly, the correlations between GBV obtained from full and reduced models reported by these authors ranged from about 0.45 to 0.95. As in our study, their reported values depended on the model used (e.g., single- vs multiple-trait) and validation strategy (e.g., tenfold cross-validation vs family validation).

The impact of environment is evident in many grass populations and can be included in the model through GxE interactions (Bornhofen et al. 2022). For models including many traits and environments as it would be the case in our study, estimates of variance components derived from multiple-trait and multiple-environment models may be unreliable, especially with limited number of records and unbalanced data across locations. An alternative is to use so-called reaction norm models (Kolmodin et al. 2002), which can produce a parsimonious model for estimating GxE interactions that allows for many environments. Reaction norm models will be tested as more data become available. Therefore, with the intention of providing reliable estimates of variance components and accurate genomic predictions such models were not considered in our current work. Still, to minimize the impact of environment on estimates of variance components and prediction of GEBV, trial location was included in the definition of contemporary group and permanent environment in the model.

## Conclusion

In the present study, genomic relationships derived from GBS data along with phenotypic records were used to estimate variance components of several traits of economic importance for a Finnish Timothy population. The moderate to large genetic variances, and the corresponding heritability, of some yield and digestibility traits showed the potential for making genetic gains in these traits. However, for the low heritable traits (e.g., winter damage), favorable correlations with other traits can be used to increase genetic gain. On the other hand, negative correlations between some yield traits and winter damage (and digestibility traits) highlight the need to use selection indices to reduce the effects of these unfavorable relationships.

The current work constituted a first step toward a routine and comprehensive genomic evaluation of Timothy in Finland. Markers obtained from GBS data were used in single- and multi-trait GBLUP to predict GEBV. While read depth and call rate directly affected the number of markers available for downstream analyses, only call rate had a considerable effect on predictive ability. Moreover, the potential benefits of using multi-trait GBLUP were not consistent and need to be further investigated. Nevertheless, the high accuracy of some yield and digestibility traits under the different validation strategies used emphasize the possibility for developing materials with improved yield and nutritional qualities.

## Supplementary Information

Below is the link to the electronic supplementary material.

Supplementary file1 (DOCX 25 KB)

## Data Availability

The data used for this study are property of Boreal Plant Breeding Ltd., and as such not publicly available.
