# Supplementary material for: Genetic architecture and genomic prediction for yield, winter damage, and digestibility traits in timothy (Phleum pratense L.) using genotyping-by-sequencing data
Source: Theor Appl Genet. 2025 Mar 18;138(4):77. doi: 10.1007/s00122-025-04860-9 (PMC11920354; doi:10.1007/s00122-025-04860-9)
Supplement: Supplementary file 1 — Supplementary file1 (DOCX 25 KB) [file 122_2025_4860_MOESM1_ESM.docx]

**Supplemental File for:**

**Genetic architecture and genomic prediction for yield, winter damage, and digestibility traits in timothy (*Phleum pratense* L.) using genotyping-by-sequencing data**

Vargas Jurado N.^1*^, Kärkkäinen H.^1^, Fischer D.^1^, Bitz, O.^1^, Manninen O.^2^, Pärssinen P.^2^, Isolahti M., Strandén I.^1^, and E. A. Mäntysaari^1^.

^1^Natural Resources Institute Finland, Luke, Jokioinen, Finland, FI-31600.

^2^Boreal Plant Breeding Ltd., Jokioinen, Finland, FI-31600.

*Corresponding author: [napoleon.vargas@luke.fi](mailto:napoleon.vargas@luke.fi), ORCID: <https://orcid.org/0000-0001-8645-3753>.

**Table S1.** Marginal means for the effect of minimum read depth and validation strategy on predictive ability^1^, defined as the correlation between GEBV and adjusted phenotypes, for yield, winter damage, and digestibility traits in Timothy. Standard errors in parentheses.

| **Trait** | **Minimum read depth** | **Family cross-validation** | **Forward prediction** |
| --- | --- | --- | --- |
| Yield at first cut | 10 | 0.256 (0.008) | 0.128 (0.003) |
|  | 20 | 0.262 (0.008) | 0.136 (0.003) |
| Yield at second cut | 10 | 0.545 (0.008) | 0.443 (0.003) |
|  | 20 | 0.551 (0.008) | 0.450 (0.003) |
| Yield at third cut | 10 | 0.327 (0.008) | 0.282 (0.003) |
|  | 20 | 0.333 (0.008) | 0.289 (0.003) |
| Winter damage | 10 | 0.198 (0.008) | 0.218 (0.003) |
|  | 20 | 0.203 (0.008) | 0.225 (0.003) |
| D-value at first cut | 10 | 0.534 (0.008) | 0.348 (0.003) |
|  | 20 | 0.540 (0.008) | 0.356 (0.003) |
| D-value at second cut | 10 | 0.613 (0.008) | 0.525 (0.003) |
|  | 20 | 0.619 (0.008) | 0.532 (0.003) |

^1^Predictive ability values averaged over the levels of maximum read depth, call rate and type of model (single- vs multiple-trait)

**Table S2.** Marginal means for the effect of maximum read depth and validation strategy on predictive ability^1^, defined as the correlation between GEBV and adjusted phenotypes, for yield, winter damage, and digestibility traits in Timothy.

| **Trait** | **Maximum read depth** | **Family cross-validation** | **Forward prediction** |
| --- | --- | --- | --- |
| Yield at first cut | 100 | 0.266 (0.008) | 0.136 (0.003) |
|  | 200 | 0.252 (0.008) | 0.128 (0.003) |
| Yield at second cut | 100 | 0.555 (0.008) | 0.451 (0.003) |
|  | 200 | 0.541 (0.008) | 0.442 (0.003) |
| Yield at third cut | 100 | 0.337 (0.008) | 0.290 (0.003) |
|  | 200 | 0.323 (0.008) | 0.281 (0.003) |
| Winter damage | 100 | 0.208 (0.008) | 0.226 (0.003) |
|  | 200 | 0.193 (0.008) | 0.217 (0.003) |
| D-value at first cut | 100 | 0.544 (0.008) | 0.356 (0.003) |
|  | 200 | 0.530 (0.008) | 0.348 (0.003) |
| D-value at second cut | 100 | 0.623 (0.008) | 0.533 (0.003) |
|  | 200 | 0.609 (0.008) | 0.524 (0.003) |

^1^Predictive ability values averaged over the levels of minimum read depth, call rate and type of model (single- vs multiple-trait)

**Table S3.** Marginal means for the effect of call rate and validation strategy on predictive ability^1^, defined as the correlation between GEBV and adjusted phenotypes, for yield, winter damage, and digestibility traits in Timothy. Standard errors in parentheses.

| **Trait** | **Call rate** | **Family cross-validation** | **Forward prediction** |
| --- | --- | --- | --- |
| Yield at first cut | 100 | 0.192 (0.009) | 0.085 (0.003) |
|  | 90 | 0.281 (0.009) | 0.141 (0.003) |
|  | 80 | 0.288 (0.009) | 0.152 (0.003) |
|  | 70 | 0.276 (0.009) | 0.149 (0.003) |
| Yield at second cut | 100 | 0.481 (0.009) | 0.399 (0.003) |
|  | 90 | 0.570 (0.009) | 0.456 (0.003) |
|  | 80 | 0.576 (0.009) | 0.467 (0.003) |
|  | 70 | 0.564 (0.009) | 0.464 (0.003) |
| Yield at third cut | 100 | 0.263 (0.009) | 0.238 (0.003) |
|  | 90 | 0.352 (0.009) | 0.294 (0.003) |
|  | 80 | 0.359 (0.009) | 0.306 (0.003) |
|  | 70 | 0.374 (0.009) | 0.303 (0.003) |
| Winter damage | 100 | 0.134 (0.009) | 0.174 (0.003) |
|  | 90 | 0.222 (0.009) | 0.231 (0.003) |
|  | 80 | 0.229 (0.009) | 0.242 (0.003) |
|  | 70 | 0.217 (0.009) | 0.239 (0.003) |
| D-value at first cut | 100 | 0.470 (0.009) | 0.305 (0.003) |
|  | 90 | 0.559 (0.009) | 0.361 (0.003) |
|  | 80 | 0.566 (0.009) | 0.373 (0.003) |
|  | 70 | 0.554 (0.009) | 0.370 (0.003) |
| D-value at second cut | 100 | 0.549 (0.009) | 0.482 (0.003) |
|  | 90 | 0.638 (0.009) | 0.538 (0.003) |
|  | 80 | 0.645 (0.009) | 0.549 (0.003) |
|  | 70 | 0.633 (0.009) | 0.546 (0.003) |

^1^Predictive ability values averaged over the levels of minimum and maximum read depth, and type of model (single- vs multiple-trait)

**Table S4.** Marginal means for the effect of type of model and validation strategy on predictive ability^1^, defined as the correlation between GEBV and adjusted phenotypes, for yield, winter damage, and digestibility traits in Timothy. Standard errors in parentheses.

| **Trait** | **Model** | **Family cross-validation** | **Forward prediction** |
| --- | --- | --- | --- |
| Yield at first cut | Single-trait | 0.261 (0.010) | 0.131 (0.004) |
|  | Multiple-trait | 0.257 (0.010) | 0.133 (0.004) |
| Yield at second cut | Single-trait | 0.550 (0.010) | 0.430 (0.004) |
|  | Multiple-trait | 0.545 (0.010) | 0.463 (0.004) |
| Yield at third cut | Single-trait | 0.333 (0.010) | 0.295 (0.004) |
|  | Multiple-trait | 0.327 (0.010) | 0.276 (0.004) |
| Winter damage | Single-trait | 0.177 (0.011) | 0.239 (0.004) |
|  | Multiple-trait | 0.224 (0.011) | 0.204 (0.004) |
| D-value at first cut | Single-trait | 0.544 (0.010) | 0.337 (0.004) |
|  | Multiple-trait | 0.530 (0.010) | 0.367 (0.004) |
| D-value at second cut | Single-trait | 0.615 (0.010) | 0.517 (0.004) |
|  | Multiple-trait | 0.617 (0.010) | 0.540 (0.004) |

^1^Predictive ability values averaged over the levels of call rate, and minimum and maximum read depth.

**Filtering criteria used in FreeBayes**

--min-base-quality10 -- min-supportingallele- qsum 10 - -read-mismatch-limit 3 - -min-coverage 5 - -no-indels - -min-alternatecount 4 - -exclude-unobserved-genotypes - -genotype-qualities - -nomnps - -no- complex - -mismatch-base-quality-threshold 10
